# Toxicity of bisphenol A (BPA) and its derivatives in divers biological models with the assessment of molecular mechanisms of toxicity

**DOI:** 10.1007/s11356-023-27747-y

**Published:** 2023-05-22

**Authors:** Kamila Rybczyńska-Tkaczyk, Bartosz Skóra, Konrad A. Szychowski

**Affiliations:** 1 Department of Environmental Microbiology, The University of Life Sciences, Leszczyńskiego Street 7, 20-069 Lublin, Poland; 2 Department of Biotechnology and Cell Biology, Medical College, University of Information Technology and Management in Rzeszow, Sucharskiego 2, 35-225, Rzeszow, Poland

**Keywords:** Endocrine disruptors, Cytotoxicity, Genotoxicity, Biotoxicity, Bisphenol A

## Abstract

The aim of the study was to determine totoxicity of bisphenol A (BPA) and its derivatives (bisphenol S (BPS), bisphenol F (BPF), and tetrabromobisphenol A (TBBPA)) due to its high accumulation in environment. The performed analysis revealed the toxicity of the BPA, BPF, and BPS against *Kurthia gibsoni*, *Microbacterium sp.*, and *Brevundimonas diminuta* as the most sensitive, reaching microbial toxic concentrations in the range of 0.018–0.031 mg ∙ L^−1^. Moreover, the genotoxicity assay shows the ability of all tested compounds to increase in the β-galactosidase level at the concentration range 7.81–500 µM (in *Escherichia coli*, PQ37). In turn, the matbolic activation of tested bishpenols has caused the enhacement of the genotoxicity and cytotoxicity effect. Interestingely, the highest phytotoxicity effect was pointed for BPA and TBBPA at the concentrations of 10 mg ∙ L^−1^ and 50 mg ∙ L^−1^, which cause the inhibition of root growth by 58% and 45%, respectively (especially for *S. alba* and *S. saccharatum*). Furthermore, the cytotoxicity analyses show the ability of BPA, BPS, and TBBPA to significantly decrease the metabolic activity of human keratynoctes in vitro after 24 h of treatment at the micromolar concentrations. Simialry, the impact of the certain bisphenols on proliferation-, apoptosis-, and inflammation-related mRNA expression was shown in tested cell line. Summarizing, the presented results have proved that BPA and its derrivatives are able to show high negative effect on certain living orgnisms such as bacteria, plants, and human cells, which is strict related to pro-apoptotic and genotoxic mechanism of action.

**Supplementary Information:**

The online version contains supplementary material available at 10.1007/s11356-023-27747-y.

## Introduction

The worldwide production of BPA has gradually increased and reached 6.2 million tons (MT) in 2020, with predicted 7.1 MT by 2027 (Staples et al. 2010; Abraham and Chakraborty 2020). Humans are exposed to BPA via dietary and non-dietary sources, e.g., BPA is used in the production of polycarbonate plastics and epoxy resins, paper products (e.g., thermal receipts), toys, medical equipment, and electronics, as well as food plastic containers, whose damage causes BPA release into stored food (Filardi et al. 2020). BPA is characterized by lipophilic properties, which facilitates penetration of biological membranes and cell interiors, and accumulation in adipose tissue (Fernandez et al. 2007). This specific property also allows to overcome both the placental barrier and blood–brain barrier; therefore, BPA and its derivatives accumulate in the fetal compartment after repeated maternal exposure (Doerge et al. 2011; Grandin et al. 2018; Filardi et al. 2020). Different concentrations of bisphenols, mainly BPA, have been detected in urine (0.45–0.73 ng mL^−1^) and serum (0.5–16 ng mL^−1^) in individuals of different ages, occupations, and gender around the world, with higher level of BPA in serum was observed in exposed workers (0.95–3.81 ng mL^−1^ and 0.48–3.19 ng mL^−1^, respectively) (Ribeiro-Varandas et al. 2013; Bousoumah et al. 2021; Xing et al. 2022).

BPA is released into the environment with effluents from wastewater treatment plants associated with the use of BPA in polycarbonate/epoxy resins (Melcer and Klecka 2011) and in untreated landfill leachates (Masoner et al. 2014). The concentration of bisphenols detected in the aquatic environment often exceeds 1500 ng L^−1^ recommended by the European Union as the predicted no-effect concentration (PNEC) (EU 2008). Bisphenol concentrations in industrial areas in Korea, China, Japan, and India can reach several times higher values (up to 7200 ng L^−1^) than in other regions (Morales et al. 2020; Cosentino et al. 2022). Bisphenols are mostly detected in the aquatic environment, where, depending on analytical detection methods, their concentrations are in the range of 1260–106,900 ng L^−1^ (lake water), 1800–107,700 ng L^−1^ (river water), and 5.3–128 ng L^−1^ (drinking water) (Gallart-Ayala et al. 2010; Yin et al. 2011; Yang et al. 2014; Shan et al. 2014; Caballero-Casero et al. 2016; Abraham and Chakraborty 2020).

Due to increased concerns regarding BPA toxicity to the environment and living organisms, its several analogs are currently being introduced as substitutes, e.g., bisphenol F (BPF), bisphenol S (BPS), and tetrabromobisphenol-A, a brominated derivate of BPA (TBBPA) (Szychowski and Wójtowicz 2013; Chen et al. 2016; Qiu et al. 2019). BPS and BPF replace BPA in many products, but TBBPA, due to its flame-retardant properties, is used as an additive to textiles, furniture, appliances, and electronic devices, especially those used in the aviation industry. Moreover, TBBPA, BPA, and its derivates are released from plastics during their use (Kousaiti et al. 2020; Holmes et al. 2021). However, these BPA derivatives also raise concerns regarding their toxicity to human health and widespread occurrence in the environment via bioaccumulation and biomagnification (Ruan et al. 2015; Chen et al. 2016; Wang et al. 2017; Zhang et al. 2018b). Recent studies have indicated that similarly to BPA, its derivatives also exert adverse effects on human health as EDs and can be accumulated in adult and fetal human tissues (Chen et al. 2016). To date, a number of health safety studies involving bisphenol analogs have shown their various toxic effects, including cytotoxicity, neurotoxicity, reproductive toxicity, genotoxicity, and carcinogenicity (Mustieles and Fernández 2020; Xing et al. 2022). Moreover, TBBPA can be microbiologically transformed into BPA in the natural environment; thus, its biologically active intermediate metabolites are still present in the environment (Szychowski and Wójtowicz 2013).

In addition to the effect on human health, BPA and its derivatives (BPS, BPF, and TBBPA) also impair the microbiological and biochemical soil balance, as well as the growth and development of plants (Xie et al. 2018; Zaborowska et al. 2019, 2020a, b, 2021; Xiao et al. 2020). Bisphenols enter agricultural soil mainly from sewage sludge and effluents from wastewater treatment plants used for agriculture irrigation, which are characterized by a significant content of these xenoestrogens (2.16–1.740 pg g^−1^ dw and 0.24–696 ng L^−1^, respectively) (Lee et al. 2015; Caballero-Casero et al. 2016; Pérez et al. 2017; Liu et al. 2021). The biotic stress triggered by the presence of bisphenols in soil causes varied responses of soil microorganisms, and thus their soil enzyme activities (Zaborowska et al. 2021). Zaborowska et al. (2019; 2020a, 2020b, 2021) indicated that BPA, BPF, and BPS interfered with the growth of soil microorganisms, especially bacteria, and disturbed their homeostasis by inhibiting enzymatic activity (Zaborowska et al. 2019, 2020a, 2021).

The aim of our study was to determine the bio-, phyto-, and genotoxicity of BPA and its derivatives (BPS, BPF, and TBBPA). Among human tissues, skin is most likely to be exposed to the external environment. Therefore, the cytotoxicity and molecular mechanism of action of these xenoestrogens on human keratinocyte HaCaT cells was also determined by evaluating the expression of genes responsible for cell growth and proliferation (*Ki67*, *ATM*, *p53*) and oxidative stress (*SOD2*, *CAT*, *SHH*, *PPARγ*, *NF-κB1*, *NRL2L2*).

## Materials and methods

### Chemicals

Bisphenol A (cat. 239658), bisphenol F (cat. 51453), bisphenol S (cat. 43034), tetrabromobisphenol A (cat. 330396), trypsin, penicillin/streptomycin, and resazurin sodium salt were purchased from Sigma-Aldrich (Germany). Phosphate-buffer saline (PBS) and Dulbecco’s modified Eagle medium (DMEM) without phenol red were obtained from Corning (USA). Charcoal–dextran treated fetal bovine serum (FBS), Gene MATRIX Universal RNA Purification Kit, and Fast Probe qPCR Master Mix (2x) plus ROX Solution were purchased from EURx (Poland). The High-Capacity cDNA Reverse Transcription Kit, TaqMan probes and primers complementary to genes encoding *ACTB* (Hs01060665_g1), *KI67* (Hs04260396_g1), *TP53* (Hs01034249_m1), *ATM* (Hs00175892_m1), *SHH* (Hs00179843_m1), *SOD2* (Hs00167309_m1), *CAT* (Hs00156308_m1), *PPARG* (Hs00234592_m1), *NFL2E2* (Hs00975961_g1), and *NFKB1* (Hs00765730_m1) were purchased from Thermo Fisher Scientific (USA). The MARA assay, Phytotoxkit, and SOS Chromotest were purchased from Tigret (Tigret, Poland distribution).

The tested compounds were purchased at the highest possible purity (certificates of analysis available at the producer’s website — Sigma-Aldrich) as a powder, which subsequently were weight and dissolved in the DMSO to obtain 1000-time concentrated stocks. These stocks were subsequently used in the analysis with the following bisphenol dilutions ranges: 0.0156–1 mg L^−1^ (MARA toxicity assay); 10 and 50 mg L^−1^ (Phytotoxkit assay), 7.81–500 µM (genotoxicity SOS Chromotest), and 1 nM–100 µM (human cell culture studies: resazurin reduction assay and qPCR). Dilutions of bisphenols tested in the present study were selected on the basis of literature data and optimization experiments.

### Assessment of toxicity using multi-species microbial assay (MARA)

The multi-species microbial assay (MARA) was used to toxicity of bisphenol derivatives, respectively. In the MARA assay, lyophilized microorganisms placed in row H of the microplates were rehydrated and pre-incubated for 4 h at 30 °C. Series of six dilutions of the initial solution of each bisphenol derivatives (1, 0.5, 0.125, 0.0625, 0.0312, and 0.0156 mg L^−1^) were placed in rows G-B of the microplates. Pure medium was introduced into row A as a strain control. Subsequently, microorganisms from row H were added to each sample dilution. Microplates were incubated at 30 °C (18 h) and subsequently scanned in a flatbed scanner (Epson Perfection V550 Photo). The results were processed using an image analysis program that facilitates calculation of the average MTC and minimum MTC (microbial toxic concentration) values (mg L^−1^). Moreover, based on the growth inhibition results for each strain, the EC_50_ value (half-maximal effective concentration) was calculated for each bisphenol and classified as: EC_50_ < 1 mg L^−1^ — very toxic to aquatic organisms, 1–10 mg L^−1^ — toxic to aquatic organisms, 10–100 mg L^−1^ harmful to aquatic organisms, and > 100 mg L^−1^ — not classified as harmful to aquatic organisms (European Commission (EC) 1993).

### Assessment of genotoxicity

For the SOS ChromoTest assessing genotoxicity, overnight bacterial cultures were grown in fresh LB medium to an optical density (OD_600nm_) of 0.5–0.6, diluted tenfold in double strength LB medium (20 g tryptone L^−1^, 10 g yeast extract L^−1^, 20 g sodium chloride L^−1^, pH 7.4), and mixed (v/v) with the tested compounds, e.g., potential mutagens (or promutagens when lyophilized rat liver S9 fraction with cytochrome P450 activity was added) and solvents. As positive controls, 4-nitroquinoline 1-oxide (4NQO), in the range from 0.078 to 10 µg mL^−1^ (without metabolic activation by S9 fraction), and 2-aminoanthracene (2AA) (with metabolic activation by S9 fraction), in the range from 0.78 to 100 µg mL^−1^, were used. A negative control (distilled water) was included in each assay. Bacteria were exposed to different concentrations of bisphenol derivatives (7.81, 15.62, 31.25, 62.50, 125, 250, and 500 µM) for 1.5 h at 37 °C. The colorimetric reactions for β-galactosidase (β-gal) and alkaline phosphatase (AP) were estimated spectrophotometrically in 96-well plates at A_600nm_ and A_420nm_. Significant genotoxic activity was defined as an adjusted induction factor (CIF) equal to or greater than 1.2.

### Phytotoxicity assessment

Phytotoxkit (Tigret, Poland) was used to determine the direct effects of bisphenol derivatives (at concentrations of 10 and 50 mg L^−1^) on the germination and growth of young roots of *Lepidium sativum*, *Sinapis alba*, and *Sorgoum saccharatum* compared to controls (distilled water) in reference soil. The germination index (GI) and root growth inhibition (RGI) of seeds exposed to bisphenols were assessed and compared with germination and growth of control, according to the manufacturer’s protocol.

### In vitro* toxicity to human cells*

#### Cell culture and treatment

The human keratinocyte cell line (HaCaT; ATCC CRL-2404) was obtained from the American Type Culture Collection (ATCC, distributor: LGS Standard, Łomianki, Poland). The cells were cultured in DMEM without phenol red, supplemented with 10% FBS and penicillin and streptomycin, in a humidified atmosphere with 5% CO_2_ at 37 °C until confluence. Subsequently, the cells were trypsynized and seeded in 96-well plates at a density of 4.5 × 10^3^ cells/well or in 12-well plates at a density of 9 × 10^4^ cells/well for the resazurin reduction assay and real-time PCR, respectively. After 24 h, the medium was removed and replaced with fresh one containing charcoal–dextran-treated FBS (known to be free of steroid hormones and various other substances) and increasing concentrations (in the range from 1 nM to 100 µM) of BPA, BPF, BPS, and TBBPA.

#### Resazurin reduction assay

The resazurin reduction assay was performed according to the protocol of Ivanov et al. (Ivanov et al. 2014). Briefly, the cells were seeded as described above and initially cultured for 24 h. Next, the medium was replaced with fresh one containing increasing concentrations (1 nM–100 µM) of BPA, BPF, BPF, and TBBPA for 24 h. Subsequently, the medium was removed and new one with 1% resazurin was added to each well for 1 h. After this time, the measurements were performed at *λ*_ex._ = 530 nm and *λ*_em._ = 590 nm using a microplate reader (FuilterMax F5). The results were normalized to the vehicle-treated cells and expressed as percentage (%) of control.

#### Real-time polymerase chain reaction (RT-PCR) in HaCaT cells

Real-time PCR was performed as described by Skóra et al. (2022). Briefly, the cells were seeded in 12-well plates 24 h before the experiment. After this time, the medium was replaced with fresh one containing 50 µM BPA, BPF, BPF, and TBBPA for 24 h. Subsequently, isolation of total RNA was performed, using the Gene MATRIX Universal RNA Purification Kit according to the producer’s manual (EURx, Poland). The quantity and quality of the RNA were assessed spectrophotometrically at 260/280 nm (ThermoFisher NanoDrop, USA). A reverse transcription reaction was then performed using the High-Capacity cDNA Reverse Transcription Kit (ThermoFisher, USA). Subsequently, the obtained cDNA template (1 µL) was used in real-time PCR together with the Fast Probe qPCR Master Mix (2x), plus ROX Solution (EURx), primers and TaqMan probes complementary to the sequences coding for the *ACTB*, *KI67*, *TP53*, *ATM*, *SHH*, *SOD2*, *CAT*, *PPARG*, *NFL2E2*, and *NFKB1* genes; total reaction volume was always 20 µL. The qPCR program used in this study was as follows: 2 min at 50 °C and 10 min at 95 °C, followed by 45 cycles of 15 s at 95 °C and 1 min at 60 °C. The threshold value (C_t_) for each sample was calculated during the exponential phase, and ΔΔCt was used to determine the average fold expression changes of the genes tested. *ACTB* was used as the reference gene.

### Statistical analysis

The data are presented as means ± SD of three independent experiments (*n* = 3). The data were analyzed using one-way analysis of variance (ANOVA) followed by Tukey’s multiple comparison post hoc test ***P < 0.001, **P < 0.01, and *P < 0.05 compared to control.

## Results

### Biotoxicity

**Fig. 1:**
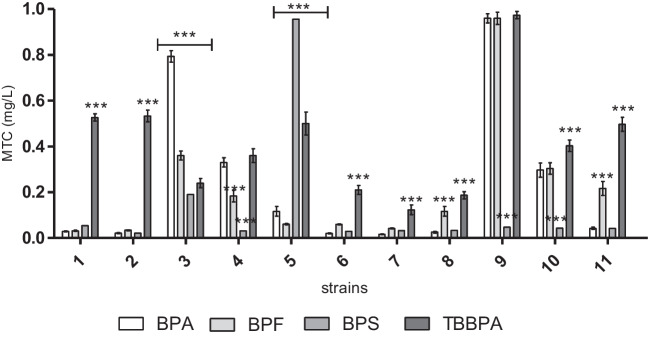
Microbial toxic concentration (MTX in mg/L) of BPA and its derivatives for each strain (1) *Microbacterium* sp., (2) *Brevundimonas diminuta*, (3) *Citrobacter freudii*, (4) *Comamonas testosteroni*, (5) *Entrococcus casseliflavus*, (6) *Delftia acidovorans*, (7) *Kurthia gibsoni*, (8) *Staphylococcus warneri*, (9) *Pseudomonas aurantiaca*, (10) *Serriatia rudidaea*, and (11) *Pichia anomala*

The wide sensitivity range of the MARA species was observed in the presence of BPA, BPF, and BPS, with the most sensitive species in the order of: *Kurthia gibsoni*, *Microbacterium sp.*, and *Brevundimonas diminuta* (MTC min. = 0.018–0.031 mg L^−1^) (Fig. 1). The BPA, BPF, and BPS samples were characterized by very low average MTC values ranging from 0.018 to 0.12 mg L^−1^, classified as toxic compounds. The TBBPA sample was characterized by low selective toxicity, with the most sensitive strain being *Kurthia gibsoni* (MTC min. = 0.13 mg L^−1^). *Pseudomonas aurantiaca* was characterized by the highest resistance to BPA, TBBPA, and BPF of the strains included in the MARA assay (MTC max. = 0.95–0.98 mg L^−1^). The highest resistence in the presence of BPS was recorded for *Enterococcus casseliflavus* (MTC max. = 0.97 mg L^−1^) (Fig. 1). Moreover, the obtained EC_50_ values classified BPA, BPS, BPF, and TBBPA as very toxic compounds (Table 1).

**Table 1:** EC_50_ values (in mg L^−1^) for all MARA strains in the presence of BPA, TBBPA, BPF, and BPS samples

Strain no	Strain name	BPA	TBBPA	BPF	BPS
1	*Microbacterium* sp.	0.023 ± 0.007*	0.868 ± 0.058	0.042 ± 0.004	nc
2	*Brevundimonas diminuta*	0.016 ± 0.002	1.831 ± 0.125	0.045 ± 0.004	nc
3	*Citrobacter freudii*	1.854 ± 0.115	0.698 ± 0.046	0.718 ± 0.021	0.469 ± 0.037
4	*Comamonas testosteroni*	0.445 ± 0.009	0.238 ± 0.024	0.416 ± 0.029	0.017 ± 0.005
5	*Entrococcus casseliflavus*	0.213 ± 0.009	1.752 ± 0.071	0.456 ± 0.014	nc
6	*Delftia acidovorans*	0.013 ± 0.004	0.117 ± 0.005	0.052 ± 0.001	nc
7	*Kurthia gibsoni*	0.010 ± 0.002	0.237 ± 0.002	0.066 ± 0.001	0.047 ± 0.003
8	*Staphylococcus warneri*	0.011 ± 0.002	0.684 ± 0.032	0.163 ± 0.006	0.035 ± 0.008
9	*Pseudomonas aurantiaca*	7.914 ± 0.282	1.243 ± 0.080	nc	0.070 ± 0.003
10	*Serriatia rudidaea*	0.544 ± 0.038	0.803 ± 0.023	0.524 ± 0.021	0.115 ± 0.004
11	*Pichia anomala*	0.107 ± 0.001	1.819 ± 0.076	0.524 ± 0.015	0.030 ± 0.002

^*^Mean value of EC_50_ values (half maximal effective concentration) ± SD; *nc*, EC_50_ value not calculated; *BPA* (bisphenol A), *TBBPA* (tetrabromobisphenol A), *BPF* (bisphenol F), *BPS* (bisphenol S)

### Genotoxicity

The results showed high genotoxicity of the initial BPA, TBBPA, BPF, and BPS solutions and their twofold dilution (7.81–500 µM), with the CIF factor ranging from 2.63 to 4.03. The genotoxicity of bisphenols in the presence of S9 fraction was indicated by *CIF* = 0.89–1.75, with a higher CIF for 7.81–250 µM concentrations (Table 2).

**Table 2:** Genotoxicity of BPA, TBBPA, BPF, and BPS with/without liver metabolism induced by rat liver S9

Sample concentrations (µM)	CIF							
	BPA		TBBPA		BPF		BPS	
	− S9	+ S9	− S9	+ S9	− S9	+ S9	− S9	+ S9
500	**2.76 (± 0.33)**	1.18 (± 0.18)	**3.51 (± 0.36)**	1.01 (± 0.12)	**2.88 (± 0.15)**	0.97 (± 0.02)	**3.17 (± 0.22)**	0.89 (± 0.09)
250	**2.63 (± 0.20)**	**1.62 (± 0.02)**	**2.89 (± 0.14)**	1.14 (± 0.09)	**3.32 (± 0.09)**	**1.28 (± 0.15)**	**3.11 (± 0.12)**	**1.34 (± 0.07)**
125	**2.86 (± 0.18)**	**1.66 (± 0.09)**	**3.16 (± 0.21)**	**1.33 (± 0.05)**	**2.60 (± 0.06)**	**1.34 (± 0.09)**	**2.95 (± 0.20)**	**1.51 (± 0.08)**
62.5	**3.96 (± 0.35)**	**1.63 (± 0.21)**	**3.61 (± 0.15)**	**1.69 (± 0.12)**	**2.46 (± 0.04)**	**1.48 (± 0.06)**	**2.02 (± 0.13)**	**1.72 (± 0.14)**
31.25	**2.55 (± 0.17)**	**1.75 (± 0.18)**	**2.17 (± 0.08)**	**1.57 (± 0.11)**	**3.93 (± 0.21)**	**1.66 (± 0.02)**	**3.23 (± 0.12)**	**1.29 (± 0.12)**
15.62	**4.03 (± 0.30)**	**1.68 (± 0.20)**	**2.74 (± 0.11)**	**1.37 (± 0.13)**	**3.05 (± 0.24)**	**1.24 (± 0.06)**	**3.01 (± 0.15)**	**1.30 (± 0.09)**
7.81	**3.15 (± 0.21)**	0.90 (± 0.01)	**2.91 (± 0.14)**	**1.24 (± 0.16)**	**3.68 (± 0.17)**	**1.21 (± 0.10)**	**3.54 (± 0.09)**	**1.23 (± 0.01)**

*CIF* – corrected induction factor, genotoxic samples are indicated in bold and shaded (CIF˃1.2), ± SD (standard deviation)

### Phytotoxicity

**Fig. 2:**
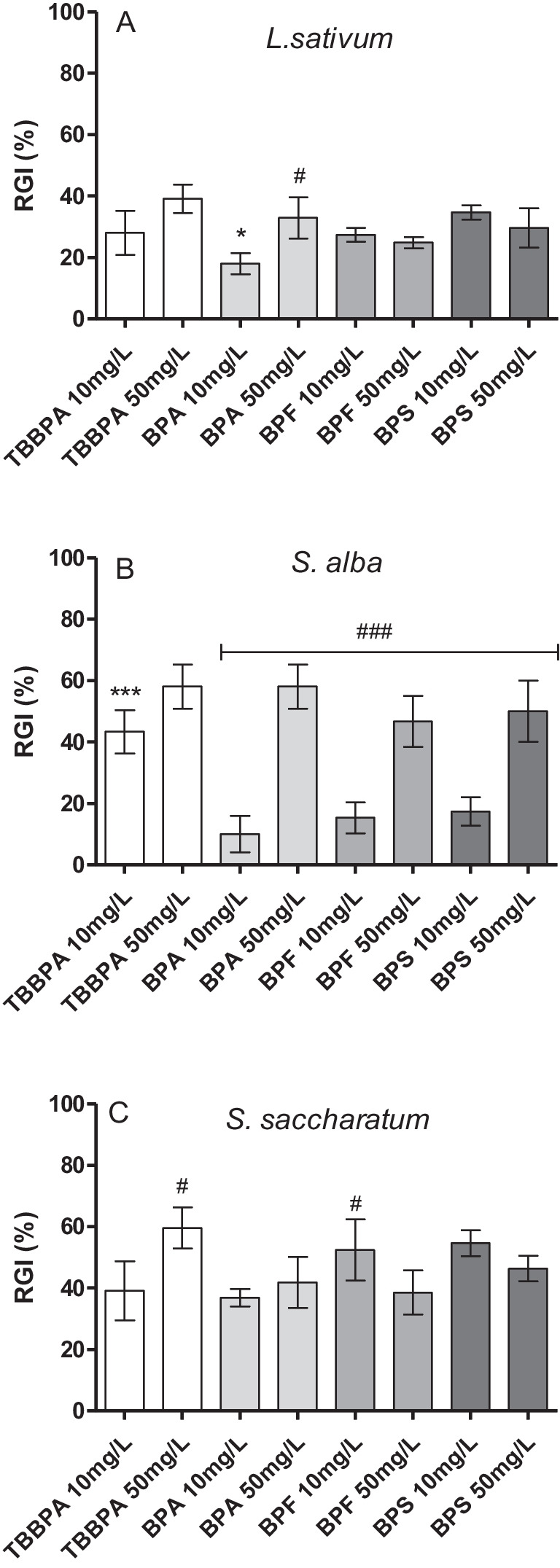
Phytotoxicity of BPA and its derivatives to *Lepidium sativum* L (**A**), *Sinapis alba* (**B**) and *Sorgoum saccharatum* (**C**); RGI, root growth inhibition; *, *** and #, ### indicate statistically significant differences between bisphenols at concentrations of 10 mg L^−1^ and 50 mg L.^−1^ at *p* < 0.05 and *p* < 0.001, respectively (one-way ANOVA, Tukey’s test)

The addition of bisphenols (BPS and BPF) did not inhibit seed germination of the test plants, and GI of *S. alba* was slightly inhibited only by 50 mg L^−1^ BPA and TBBPA (70 and 90%, respectively; Figure S1). The phytoxicity test showed the effect of bisphenols mainly on the root growth of *L. sativum*, *S. alba*, and *S. saccharatum* plants. For all test plants, a significant root growth inhibition was recorded in the presence of BPA, TBBPA, BPF, and BPS at concentrations of 10 and 50 mg^−1^, with the highest *RGI* = 58% and 44.62% in the presence of BPA and TBBPA at 50 mg L^−1^, determined for *S. alba* and *S. saccharatum*, respectively (Fig. 2).

### Cytotoxicity and metabolism of HaCaT cells

#### Metabolic activity assay

**Fig. 3:**
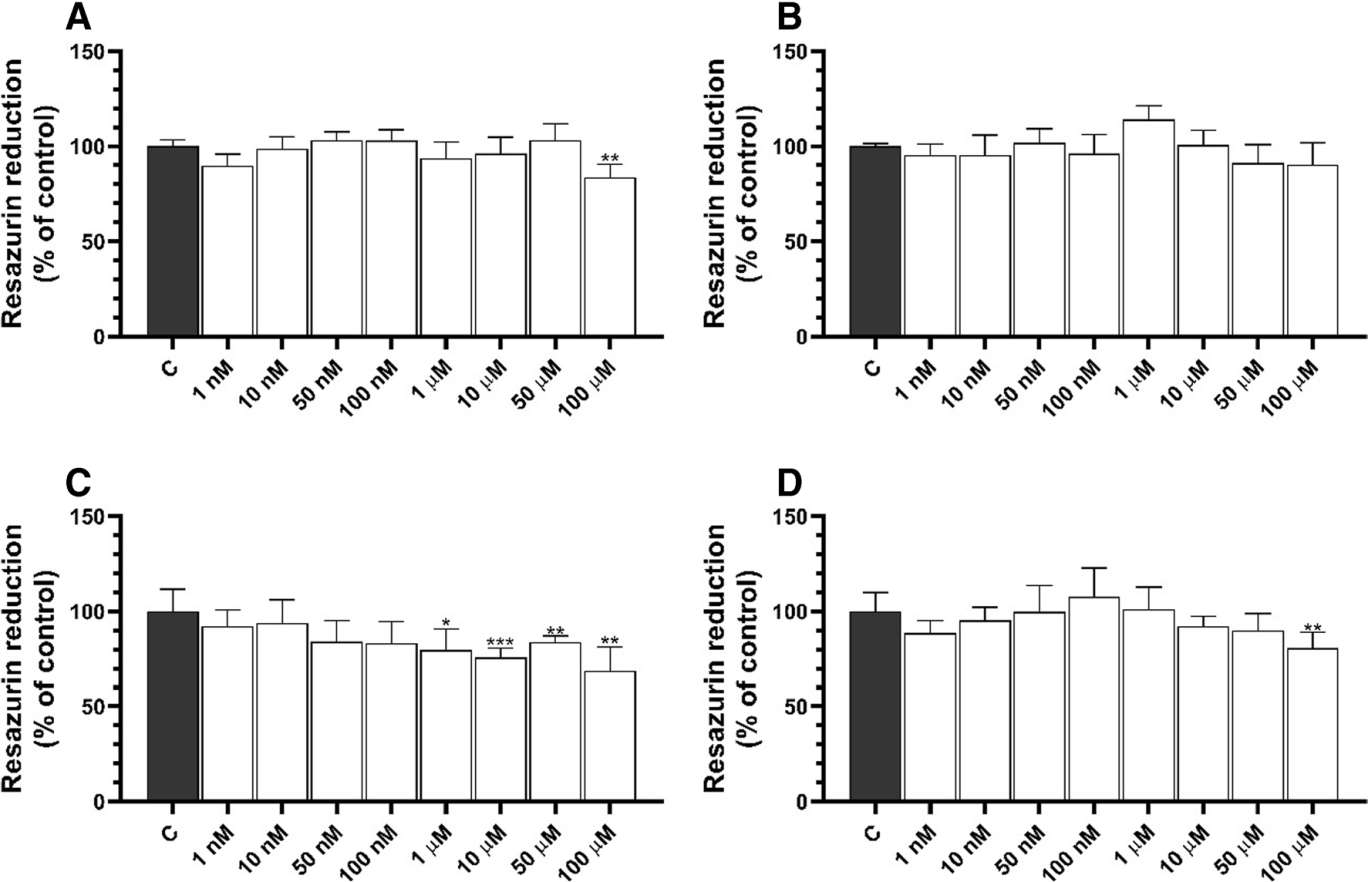
Metabolic activity of HaCaT cells treated with BPA (**A**), BPF (**B**), BPS (**C**), and TBBPA (**D**) for 24 h. Mean values (*n* = 3) with standard deviation (error bars) marked with *, **, and *** are statistically different from the respective (vehicle-treated) control at *p* < 0.05, *p* < 0.01, and *p* < 0.001, respectively (one-way ANOVA, Tukey’s test)

After 24 h of treatment, only 100 µM BPA caused a decrease in metabolic activity (by 16.39%) compared to control (Fig. 3A). No changes were observed for the other tested BPA concentrations in HaCaT cells (Fig. 3A). HaCaT cells treated with BPS for 24 h showed a decrease in metabolic activity compared to control: 20.35, 24.20, 16.04, and 31.30% for 1, 10, 50, and 100 µM BPS, respectively (Fig. 3C). In turn, only the highest TBBPA concentration caused a decrease in metabolic activity (by 19.32%) after 24-h treatment compared to control (Fig. 3D).

#### Expression of genes responsible for growth and proliferation (Ki67, ATM, p53) and oxidative stress (SOD2, CAT, SHH, PPARg, NFKβ1, NRL2L2) in HaCaT cells

**Fig. 4:**
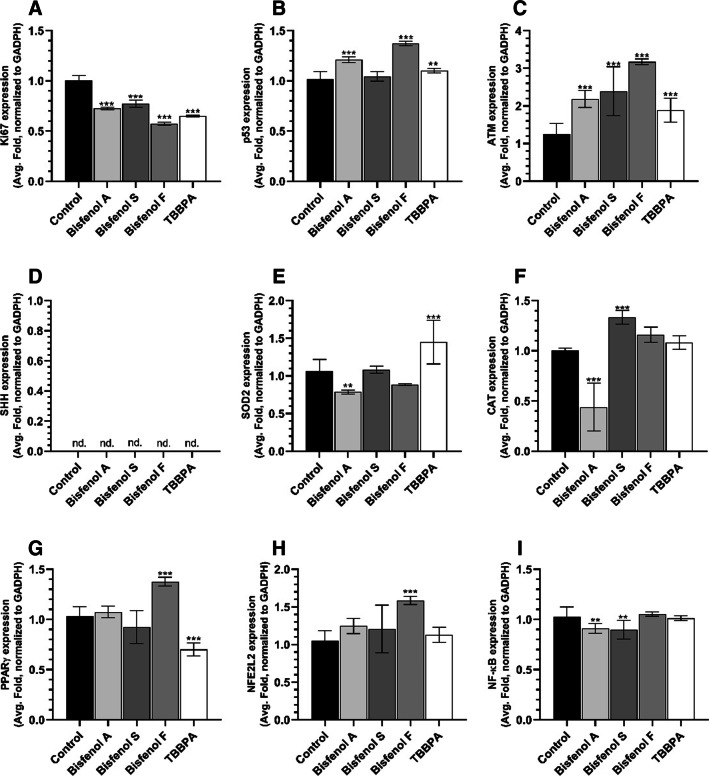
Expression of the *KI67* (**A**), *TP53* (**B**), *ATM* (**C**), *SHH* (**D**), *SOD2* (**E**), *CAT* (**F**), *PPARγ* (**G**), *NFE2L2* (**H**), and *NF-κB* (**I**) genes in HaCaT cells treated with BPA, BPS, BPF, and TBBPA for 24 h. Mean values (*n* = 3) with standard deviation (error bars) marked with *, **, and *** are statistically different from the respective (vehicle-treated) control at *p* < 0.05, *p* < 0.01, and *p* < 0.001, respectively (one-way ANOVA, Tukey’s test). ACTB was used as a reference gene; nd, gene expression not detected

After 24-h treatment of HaCaT cells, mRNA expression of *KI67* decreased by 27.45, 22.71, 42.76, and 35.04% compared to control in cells treated with BPA, BPS, BPF, and TBBPA, respectively (Fig. 4A). On the other hand, an increase in *TP53* gene expression was observed in HaCaT cells treated with BPA and BPF (by 20.90 and 37.14%, respectively) (Fig. 4B). All tested compounds caused higher *ATM* mRNA expression by 118.61, 138.99, 217.19, and 89.29% for BPA, BPS, BPF, and TBBPA, respectively, compared to control (Fig. 4C). Moreover, HaCaT cells treated with BPA and BPF showed a 21.22 and 11.53% decrease in *SOD2* expression, while TBBPA caused elevated mRNA expression by 44.91% compared to control (Fig. 4E). BPA-treated cells were characterized by a decrease in *CAT* mRNA expression by 56.01% compared to control, unlike BPS, which was able to increase this gene expression by 33.47% compared to control (Fig. 4F). The expression of *PPARγ* mRNA increased only in BPF-treated cells by 37.80%, while TBBPA caused a decrease in this gene expression by 29.83% compared to control (Fig. 4G).

## Discussion

The presence of bisphenol A and its derivatives in water and soil contributes to the disturbance of the biological balance of microorganisms and plants. The microbial strains used in the MARA assay belong to typical environmental strains naturally occurring in the soil and aquatic environment (Gabrielson et al. 2003; Bisht et al. 2010). Individual strains show different sensitivity to different chemicals and the obtained inhibition values provide a toxic fingerprint of the chemicals tested based on a panel of assays within a single test. Some of these strains, such as *Pseudomonas* sp., *Microbacterium* sp., *Comamonas* sp., or *Delftia* sp*.* are involved in the biodegradation of xenobiotics, e.g., phenols, polychlorinated bis-phenyls, and per- and polyfluorinated alkyl substances and pesticides (Singh et al. 2003; Abdel-Razek et al. 2015; Braña et al. 2016; Mishra et al. 2021; Harris et al. 2022). Moreover, previous data have indicated that rhizosphere bacteria, e.g., *Kurthia* sp., participate in naphthalene and anthracene decomposition (Bisht et al. 2010), and *Kurthia* sp. and *Delphia* sp. have the ability to promote plant growth (Bisht et al. 2010; Braña et al. 2016). Associative interactions of plants and microorganisms in soil bioremediation offer great opportunities because a suitable rhizosphere strain can be introduced together with an appropriate plant, thereby enhancing the bioremediation process. Moreover, bacteria that effectively colonize roots and decompose contaminants exploit growing roots that act as an injection system to spread the bacteria in soil (Bisht et al. 2010). Therefore, the toxic effect of xenobiotics against bacterial strains involved in the biodegradation of xenobiotics significantly reduces bioremediation efficiency of the microflora of the soil and water environment (Mishra et al. 2021). In accordance with the European Directive EC 93/67/EEC (European Commission (EC) 1993), chemicals are classified on the basis of their EC_50_ values — those with EC_50_ < mg L^−1^ are considered very toxic to aquatic organisms, 1–10 mg L^−1^ are toxic to aquatic organisms, 10–100 mg L^−1^ are harmful to aquatic organisms, and > 100 mg L^−1^ are not classified as harmful to aquatic organisms. This study showed that BPA, BPF, BPS, and TBBPA were characterized by acute toxicity for the tested microorganism, especially for *Kurthia gibsoni*, *Microbacterium* sp., and *Brevundimonas diminuta*, and based on European Directive EC 93/67/EEC, they should be classified as very toxic to aquatic organisms. On the other hand, previous studies have indicated that soil contamination with BPA, BPF, BPS, and TBBPA also poses a potential ecological risk to soil microbial community (Xie et al. 2018; Zaborowska et al. 2019, 2020b, 2021). Sources of BPA to soils include the application of sewage sludge (Langdon et al. 2012), irrigation with wastewater effluent (Chen et al. 2011), and amended with biosolids (Staples et al. 2010). The half-life of BPA in soil ranging from 3 to 37.5 days (Careghini et al. 2015) or even 75 days (Corrales et al. 2015). BPA concentrations in soil depending on the amount and type of effluent or waste received (Li et al. 2021; Dueñas-Moreno et al. 2022). Soils specifically treated with sewage sludge, irrigated with wastewater, and amended with biosolids contained BPA concentrations: 1–150 μg kg^−1^ (Langdon et al. 2012), 1.60–30.20 μg kg^−1^ (Gibson et al. 2010), and 0.25–1.15 μg kg^−1^ (Staples et al. 2010). Although the presence of BPA in agricultural fields irrigated with wastewater effluent is less than 10 μg kg^−1^ (Chen et al. 2011), Xie et al. (2018) indicated that growth inhibition of *Chloroflexi* (3.5–8.1%) and *Actinomycetes* (6.7–8.0%) was previously observed under aerobic and anaerobic conditions in the soil exposed to TBBPA (40 mg kg^−1^), but interestingly, TBBPA at this dose promoted the growth of *Proteobacteria* (35.7–41%), *Bacteroidetes* (only aerobic condition, 6.4%) and *Acidobacteria* (only under anaerobic conditions, 15.6%). This was consistent with our results, which indicated that *Pseudomonas auriantiaca* (ɤ-proteobacteria) was the most resistant strain to TBBPA contamination. Zaborowska et al. (2019, 2020a, 2020b, 2021) reported that BPA, BPF, and BPS inhibited the growth of soil microorganisms and disturbed their biochemical properties. Moreover, BPA, BPF, and BPS impaired the bioremediation potential of soil microorganisms (Zaborowska et al. 2019, 2020b, a).

In addition to the ecological balance of soil microorganisms, proper plant growth is also very important. Bisphenol A and its derivatives, in addition to disrupting the biodiversity of microorganisms in the water and soil environment, can also adversely affect plant germination and root growth (Dogan et al. 2010; Tian et al. 2014; Zhang et al. 2018a). The presence of xenoestrogens such as BPA and its derivatives in soil can significantly affect the germination and growth of crops and industrial plants, and disturb their phytoestrogen balance (Wang et al. 2015; Kim et al. 2018; Li et al. 2018a, b; Xiao et al. 2020). As described previously, BPS in soil are unstable; thus, changes in soil environmental conditions can cause the release of BPS and its metabolites from physicochemical entrapment and their subsequent accumulation in plants (Cao et al. 2020).

Seed germination is the starting point of plant life when the radicle breaks through the seed coat and develops into a new plant (Xiao et al. 2020). Previous studies indicated that different BPA concentrations can exert various effects on seed germination, e.g., BPA concentration ˃ 50 mg L^−1^ inhibited seed germination of *Cicer arietinum* (chickpea) and *Arabidopsis thaliana* (Dogan et al. 2010; Tian et al. 2014). This could be related to the inhibition of energy metabolism by higher BPA doses during seed germination (Xiao et al. 2020). In the case of chickpea, TBBPA also inhibited seed germination, but only at a dose of 100 mg L^−1^ (Dogan et al. 2010), while lower BPA concentrations (10 and 50 mg L^−1^) did not affect seed germination of these plants (Dogan et al. 2010; Tian et al. 2014). Moreover, the germination rates of various species treated with BPA differed, indicating that BPA tolerance among seeds of various plant species varied (Xiao et al. 2020).

Root growth is another parameter estimating the direct effects in plants exposed to xenobiotics. Previous studies have shown that BPA and its derivatives, depending on the exposure dose, can have different effects on plant root growth, usually induced oxidative stress (Dogan et al. 2010; Tian et al. 2014; Zhang et al. 2018a; Xiao et al. 2020; Ali et al. 2022). The production of reactive oxygen species (ROS) is a general primary response of plants to stress (Zhang et al. 2018a, b) and may involve self-protection responses of plants to stress induced by BPA exposure (Dogan et al. 2010; Zhang et al. 2018a, b). Previous studies showed that low BPA levels (1.5–50 mg L^−1^) induced ROS production in *Glycine max* (soybean) and *Cicer arietinum* (chickpea) roots. These results indicate that ROS in roots may be involved in the oxidative metabolism of BPA, which may prevent BPA damage to exposed plants. Exposure to low doses of BPA was also shown to enhance mitochondrial energy production in root cells (through the Krebs Cycle) and key enzyme activities in root respiration (Zhang et al. 2018a, b; Xiao et al. 2020). Zhang et al. (2018a, b) and Dogan et al. (2010) argued that longer BPA exposure led to higher ROS production, which in turn probably induced the expression of antioxidant enzymes degrading BPA (Dogan et al. 2010; Zhang et al. 2018a). On the other hand, Li et al. (2018a) indicated that BPA exposure at doses 6 and 17.2 mg L^−1^ could inhibit roots growth of *Glycine max L.* at 14.4 and 34.7%, respectively (Li et al. 2018a).

In addition to acute toxicity, it is also important to determine the long-term effects of xenobiotics, e.g., genotoxicity. While food safety and health organizations consider BPA a class 2 B reproductive toxin, the available data do not show a clear link between BPA and genotoxicity and carcinogenicity (Jalal et al. 2018; Ramos et al. 2019). Previous studies have not clearly confirmed BPA’s genotoxicity, but some of them indicated that BPA directly interfered with the mechanisms of cell division (George et al. 2008) and expression of genes involved in mitotic processes (Ribeiro-Varandas et al. 2013). Some data indicated genotoxic effects of BPA associated with decreased cellular proliferation at concentrations ≥ 100 μM (Bolli et al. 2008). However, our research showed that although the CIF factor was lower after metabolic activation using rat liver S9 fraction, bisphenols were still genotoxic. As previously described, microsomal cytochrome P450 enzymes in the liver can efficiently metabolize BPA via the glucuronidation and sulfation pathways; however, the bisphenol-o-quinone intermediate has been shown to be toxic and act as a DNA adduct (Kang et al. 2006; Jalal et al. 2018). Food and beverages are the main source of exposure to BPA and its derivatives into the human body (Genuis et al. 2012). However, the second largest source of human BPA and its derivatives exposure after food and beverage packaging is thermal paper (cash receipts, parking, airline and cinema tickets, luggage tags, bus and train tickets, and grocery weight tickets) (Reale et al. 2021). Therefore, we have examined the effect of BPA, BPS, BPF, and TBBPA cytotoxicity on HaCaT cells, as well as their impact on the expression of proliferation- and oxidative stress-related genes. Our results showed that after 24-h exposure of HaCaT cells to the test compounds, BPF did not significantly affect cell metabolism, while BPA and TBBPA inhibited resazurin reduction only at the highest, micromolar concentrations. Interestingly, BPS decreases the values of the resazurin reduction assay over a wide range of micromolar concentrations (1–100 µM). Previous data indicated that BPA could significantly affect the activity of HaCaT cells by activating DNA damage marker protein and inducing cell apoptosis. To date, the toxic effects of BPA have been well-described in various types of cell cultures and aquatic organisms, as well as mammals such as mice and rats (Jeong et al. 2013; Hwang et al. 2013; Abdel-Tawwab and Hamed 2018; Apaydin et al. 2019). Toxicity have been also reported for BPA derivatives such as BPF and BPS. Hercog et al. (2019) found that after 72 h of exposure of human hepatocellular carcinoma (HepG2) cells to 20 µg/mL BPA, BPS, and BPF, only BPS and BPF significantly reduced cell viability (Hercog et al. 2019). The latter authors reported that BPA also slightly decreased cell viability, but this effect was not statistically significantly. Huang et al. (2020) showed that 100 µM BPA, BPS, and BPF decreased the viability of the human granulosa (KGN) cell line (Huang et al. 2020). Interestingly, 100 nM BPS and BPF was shown to increase cell viability, which could also suggest their higher proliferation. In addition, TBBPA was also described to reduce cell viability in the broad range of concentrations, starting from 1 to 10 µM in mouse cortical and hippocampal neurons, 5 µM in human hepatocytes (L02), and 40 µM in the RAW264.7 cell line to 100 µM in the human choriocarcinoma-derived placental JEG-3 cell line (Wojtowicz et al. 2014; Honkisz and Wójtowicz 2015; Szychowski and Wójtowicz 2016; Park et al. 2019; Zhang et al. 2019). Therefore, our data are consistent with those described by other groups and indicate that HaCaT cells are relatively resistant to TBBPA-induced toxicity.

Based on the data from our research and literature reports, we have selected BPA, BPS, BPF, and TBBPA at 50 µM concentrations for further study. Our data showed that all compounds decreased the expression of the *KI67* gene, which is a well-established indicator of cell proliferation (Miller et al. 2018). Similarly, all studied compounds increased *ATM* mRNA expression, i.e., a recognized marker of cell stress and DNA damage (Mazan-Mamczarz et al. 2011). However, only BPA and BPF increased the expression of *TP53* mRNA, suggesting an initiated process of apoptosis. Our data showed that BPA and BPF reduced *SOD2* mRNA expression, while TBBPA increased it in the HaCaT cell line. Moreover, our experiments showed that BPA reduced the levels of *CAT* mRNA transcript, while increased *CAT* mRNA expression was observed after BFS treatment. In our study, we did not detect *SHH* transcripts in HaCaT cells.

To date, only one article has described the role of ATM in the mechanism of bisphenol action (Mahemuti et al. 2018). In normal human fetal lung fibroblasts (HFLF), 100 µM BPA has been reported to strongly increase the expression of ATM-positive cells (Mahemuti et al. 2018). Higher expression of TP53 mRNA and/or protein levels by BPA is well documented in a number of cell types. Loyd et al. (2019) found that BPA increased TP53 protein expression in the breast carcinoma T-47D cell line at a concentration range of 200 nM to 2 µM, in and in the breast adenocarcinoma MCF-7 cell line at a concentration range of 1–2 µM (Lloyd et al. 2019). Similar results were obtained by Lucendo-Villarin et al. (2020), who showed that BPA, BPS, and BPF increased *TP53* mRNA expression in the human pluripotent stem cell lines (Man12 (male)) (Lucendo-Villarin et al. 2020). Consistent results regarding elevated TP53 protein expression were also acquired in the mouse-derived C17.2 multipotent neural stem cell line treated with TBBPA at concentrations ranging from 12.5 to 50 µM (Cho et al. 2020). Moreover, contrary to our results, Hercog et al. (2019) showed that BPA, BPS, and BPF, at concentrations of 10 ng mL^−1^ and 10 µg mL^−1^, did not alter mRNA expression of *GPX1*, *SOD1A*, *CAT*, and *TP53* antioxidant enzymes in HepG2 cells; however, the latter authors tested lower concentrations of bisphenols; therefore, the effect could be dose-dependent (Hercog et al. 2019). Huang et al. (2020) reported that BPA, BPS, and BPF reduced CAT, SOD, and GSH activities at a wide range of concentrations (between 1 and 100 µM) in human granulosa KGN cells (Huang et al. 2020). Similar to bisphenols, 1.00 mg L^−1^ TBBPA decreased the expression of *SOD1*, *CAT*, and *GPX1A* mRNAs expression in embryos and larvae of zebrafish (*Danio rerio*) (Wu et al. 2016). On the other hand, TBBPA concentrations from 5 to 40 µM increased *CAT* mRNA expression in L02 cells (Zhang et al. 2019). Therefore, we could conclude that our data were largely consistent with previous reports and showed that the selected bisphenol analogs exerted genotoxic (as demonstrated by increased *ATM* gene expression) and antiproliferative activity (proved by *KI67* mRNA expression).

Nuclear factor erythroid 2-like 2 (NFE2L2) is a transcription factor that controls the cellular defense system against toxic and oxidative damage through the expression of genes involved in the oxidative stress response and drug detoxification (He et al. 2020). Similarly, peroxisome proliferator-activated receptor gamma (PPARγ) and sonic hedgehog (SHH) are involved in responses to xenobiotics, oxidative stress, cell metabolism, apoptosis, and cell differentiation (Janani and Ranjitha Kumari 2015; Lee et al. 2018; Lv et al. 2018). Therefore, to elucidate the mechanism of action of BPA, BPS, BPF, and TBBPA, we decided to analyze the levels of *PPARγ*, *NFE2L2*, *NF-κB*, and *SHH* mRNA transcripts. Our study demonstrated that BPF increased *PPARγ* mRNA level, while TBBPA reduced it. However, only BPF increased the expression of *NFE2L2* mRNA. The current study showed that all test compounds were able to modify the level of *NF-κB* transcripts, while no *SHH* mRNA expression was detected in HaCaT cells. Interestingly, Gao et al. (2020) reported that 100 µM BPA and BPS increased *PPARγ* mRNA levels in the human macrophage THP-1 cell line (Gao et al. 2020), while 25 µM BPA and BPS was sufficient to increase *PPARγ* mRNA expression in murine 3T3-L1 preadipocytes (Ahmed and Atlas 2016); however, the latter authors did not observe changes in *PPARγ* mRNA at lower concentrations (0.01 to 10 µM) of the studied compounds (Ahmed and Atlas 2016). In human adipose-derived stem cells, BPS and BPF were shown to increase *PPARγ* mRNA in the range of 1–25 µM and 10–25 µM for BPS and BPF, respectively, and promote lipid accumulation and adipogenesis (Reina-Pérez et al. 2021). It has been well documented that TBBPA and its derivatives are able to increase *PPARγ* mRNA expression in the 3T3-L1 cell line (Riu et al. 2011). Moreover, Wójtowicz et al. (2014) reported that TBBPA acted through the PPARγ pathway in primary mouse neocortical neurons and observed decreased levels of these receptors (Wojtowicz et al. 2014). Therefore, our data are consistent with the current state of knowledge. Moreover, it was demonstrated that 10 and 50 µM TBBPA increased NFE2L2 protein levels in the human hepatoma Hep3B cell line, which, according to these authors, indicated the mechanism of antioxidant enzyme expression control (Oguro et al. 2021). Unfortunately, data concerning NFE2L2 expression after BPS and BPF treatments are not available. It was shown that TBBPA-induced oxidative stress and apoptosis in L02 cells was mediated through the *NFE2L2* signaling pathway (Zhang et al. 2019). Interestingly, TBBPA concentrations of 12.5 to 50 µM decreased NFE2L2 protein expression in C17.2 cells (Cho et al. 2020). It was also reported that 10 nM BPA enhanced the NF-κB-IL-6 signaling pathway in the SH-SY5Y cell line and increased its malignancy (Xiong et al. 2017). An increase in NF-κB protein expression was observed also in primary macrophages isolated from red common carp following 100 µg L^−1^ BPA treatment (Yang et al. 2015). Moreover, BPS and BPF elevated *NF-κB* mRNA expression at concentration ranges of 100–1000 µg L^−1^ and 10–1000 µg L^−1^, respectively (Qiu et al. 2018). Guan et al. (2021) showed that nanomolar concentrations of TBBPA were sufficient to increase NF-κB protein expression in the human Ihikawa endometrial carcinoma cell line. Similarly, in the mouse macrophage RAW 264.7 cell line, TBBPA concentration in the range of 10–50 µM increased NF-κB levels (Han et al. 2009). The involvement of the SHH gene in the mechanism of action of BPA, BPS, BPF, and TBBPA is mainly unknown. The only available data showed that BPA in the human adrenocortical H295A cell line activated the SHH signaling pathway and increased its expression via estrogen receptor beta (Erβ) (Medwid et al. 2018).

## Conclusions

Summarizing, presented results clearly show that the tested bisphenol analogs (BPA, BPS, BPF, and TBBPA) are characterized by high negative mechanism of action on microorganisms, plants, and cell proliferation. The observed effect was strict correlated with the genotoxicity, direct impact on metabolism as well as on mRNA expression on genes related to the proliferation, apoptosis, and inflammation in keratinocytes in vitro. Moreover, our results showed high genotoxicity of BPA, TBBPA, BPF, and BPS before and after metabolic activation with rat liver S9 fraction. Furthermore, the tested bisphenols were able to significantly inhibit the growth of the tested microorganisms (bacteria and yeast). The phytotoxicity test showed that bisphenols mainly inhibited root growth, proving the negative impact of such substance on root system. Our study sheds new light on the negative effect of bisphenol compounds on environment, which is of high importance considering their high content in daily care skin products.

## Supplementary Information

Below is the link to the electronic supplementary material.

Supplementary file1 (DOCX 52 KB)

## Data Availability

Original data available for request.

## Abbreviations

ACTB: Beta-actin
ATM: Ataxia telangiectasia mutated
β-gal: β-Galactosidase
BPA: Bisphenol A
BPF: Bisphenol F
BPS: Bisphenol S
DMEM: Dulbecco’s modified Eagle medium
FBS: Fetal bovine serum
KI67: Antigen Ki-67
TBBPA: Tetrabromobisphenol A
CAT: Catalase
CIF: Corrected induction factor
GI: Germination index
PPARγ: Peroxisome proliferator-activated receptor gamma
p53: Protein 53
RGI: Root growth inhibition
ROS: Reactive oxygen species
SHH: Sonic hedgehog homolog
SOD: Superoxide dismutase
2AA: 2-Aminoanthracene
4NQO: 4-Nitroquinoline 1-oxide
