# Supplementary material for: Toxicity of bisphenol A (BPA) and its derivatives in divers biological models with the assessment of molecular mechanisms of toxicity
Source: Environ Sci Pollut Res Int. 2023 May 22;30(30):75126–40. doi: 10.1007/s11356-023-27747-y (PMC10293331; doi:10.1007/s11356-023-27747-y)
Supplement: Supplementary file 1 — Supplementary file1 (DOCX 52 KB) [file 11356_2023_27747_MOESM1_ESM.docx]

Figure 1S. Germination index (GI) of BPA and its derivatives against *Lepidium sativum* L (A), and *Sinapis alba* (B) and *Sorgoum saccharatum* (C)
